# “The system has to be health literate, too” - perspectives among healthcare professionals on health literacy in transcultural treatment settings

**DOI:** 10.1186/s12913-021-06614-x

**Published:** 2021-07-21

**Authors:** Annika Baumeister, Digo Chakraverty, Angela Aldin, Ümran Sema Seven, Nicole Skoetz, Elke Kalbe, Christiane Woopen

**Affiliations:** 1 Cologne Center for Ethics, Rights, Economics, and Social Sciences of Health (CERES), University of Cologne and Research Unit Ethics, Faculty of Medicine and University Hospital Cologne, 50931 Cologne, Germany; 2 Medical Psychology | Neuropsychology and Gender Studies & Center for Neuropsychological Diagnostics and Intervention (CeNDI), Faculty of Medicine and University Hospital Cologne, University of Cologne, 50937 Cologne, Germany; 3 Evidence-Based Internal Medicine, Department I of Internal Medicine and Center for Integrated Oncology Aachen Bonn Cologne Duesseldorf, Faculty of Medicine and University Hospital Cologne, University of Cologne, 50935 Cologne, Germany

**Keywords:** Organizational health literacy, Migration, Health communication, Ethnic concordance, Qualitative research

## Abstract

**Background:**

Effective communication is a central aspect of organizational health literacy. Healthcare professionals are expected to ensure an effective and satisfactory flow of information and to support their patients in accessing, understanding, appraising, and applying health information. This qualitative study aimed to examine the health literacy-related challenges, needs, and applied solutions of healthcare professionals when engaging with persons with a migrant background. Based on the integrated model of health literacy (Sørensen et al., BMC Public Health 12:80, 2012), we focused on environmental, personal, and situational factors that shape health literacy in transcultural treatment settings.

**Methods:**

We conducted five focus group discussions with healthcare professionals (*N* = 31) who are in regular contact with persons with a migrant background. Discussions were transcribed verbatim and analyzed using qualitative content analysis by applying a deductive–inductive categorization procedure. Deductive categories were derived from the integrated model of health literacy.

**Results:**

Challenges included a mismatch in the provision and use of health services. Participants regarded easily accessible services and outreach counselling as helpful solutions. Further challenges were the migrant patients’ distrust in healthcare professionals and the German healthcare system, the participants’ uncertainty in dealing with patients’ expectations and needs, and the patients’ non-compliance with appointments. Environmental factors included systemic lack of time and economic pressure. Both were reported as impeding the flow of information in all treatment settings. Participants with a migrant background themselves (*n* = 16) regarded this personal factor as an opportunity that increased patients’ trust in them. They also reported challenges such as high levels of responsibility felt when ad hoc interpreting for colleagues.

**Conclusions:**

Known issues observed in the delivery of healthcare for the majority population (i.e., systemic lack of time, economic pressure) appear to be intensified in the context of migration. An increasingly diverse patient clientele indicates a growing need for culture-sensitive, health-literate healthcare organizations. A corresponding diversity of the health workforce is desirable and should be strengthened by national finance and educational programs. Healthcare professionals who interpret for colleagues should be given the necessary time. Further studies are needed to develop appropriate interventions for improving health literacy at individual and organizational levels. Funding for interpreting services should be expanded.

**Supplementary Information:**

The online version contains supplementary material available at 10.1186/s12913-021-06614-x.

## Background

In recent years, the steady increase in international migration has resulted in new discussions regarding the challenges and responsibilities for European host countries. These include, but are not limited to, ethical debates on humane and equitable living conditions, human rights and equal opportunities for newly arrived immigrants. It has also raised particular issues for health systems in responding fast to the growing healthcare needs of diverse immigrant populations.

**Fig. 1:**
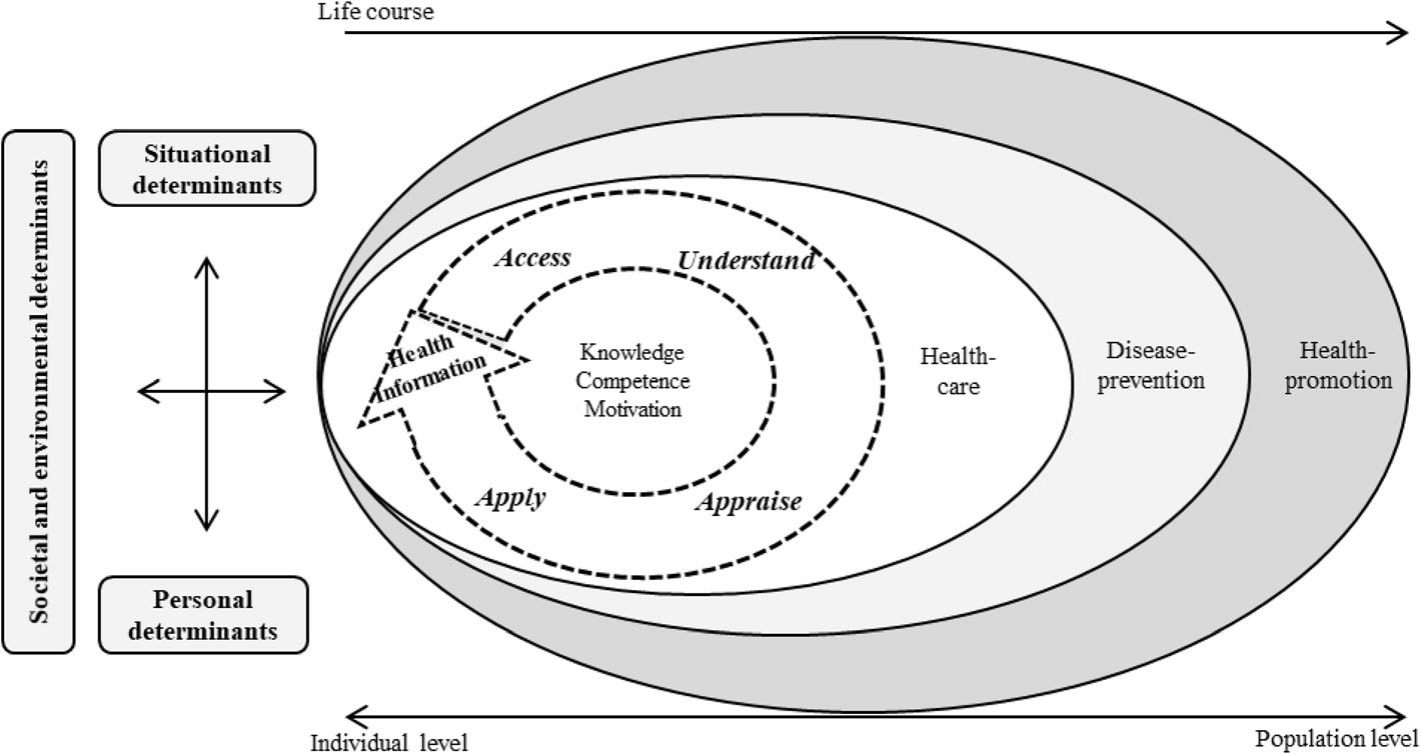
Simplified version of the integrated model of health literacy. Adapted from the Sorensen et al. (2012) [3].

Over the last two decades, the concept of health literacy has increasingly become the focus of health research and policy, including the development of various international initiatives and national action plans to improve health literacy at the individual and population levels [1]. Initially, health literacy was defined as a rather narrow, educational concept that linked literacy and numeracy skills to the abilities required to understand and use health-related information in the medical setting [2]. Over time, this gradually evolved into a multidimensional construct referring to a broadly defined set of individual (cognitive, motivational, and social) resources, skills, and abilities, which are closely interrelated with situational factors and environmental conditions, such as the requirements of the health system [3, 4]. According to the European Health Literacy Consortium “*Health literacy is linked to literacy and entails people’s knowledge, motivation and competences to access, understand, appraise, and apply health information in order to make judgments and take decisions in everyday life concerning healthcare, disease prevention and health promotion to maintain or improve quality of life during the life course.”* [3] (p. 3). Based on this definition, the researchers derived the integrated model of health literacy, which emphasizes the social-relational character of health literacy by including the personal, societal, environmental, and situational factors that influence an individual’s health literacy over the life course. Figure 1 presents a simplified version of the integrated model of health literacy.

Migration can thereby be understood as either a personal determinant of health literacy, i.e., having a migrant background or a situational factor, i.e., experiences of migration and being confronted with an unknown health system. Both are interrelated with societal, i.e., cultural and environmental aspects such as the health literacy responsiveness of the receiving country’s health system. All these factors may influence how individuals acess information and how transcultural interactions in health systems are shaped.

European population studies on health literacy indicate that persons with a migrant background, i.e., those who either migrated themselves to another country or whose parents did so, have comparatively more difficulties in accessing, understanding and using health information than the general population [5–7]. In Germany, this was true for 71% of people with a migrant background, compared to 52.8% of the majority non-migrant population [8]. These studies align with other empirical findings, indicating that migrants and people from ethnic minorities are at high risk of low health literacy [9–12] as well as social and health-related inequalities [13–15].

Recent approaches in health literacy research and policy have been characterized by the development of more system-oriented strategies that particularly focus on the responsibility of health systems and healthcare organizations to develop, maintain and promote individual and population health literacy [16]. The promotion of organizational health literacy includes, in particular, the establishment of health-literate healthcare organizations. These should, among other factors, adequately respond to the individual needs of diverse populations with varying health literacy skills, avoid the stigmatization of such populations, promote successful interpersonal communication and ensure equitable access to reliable health information [17, 18].

Healthcare professionals represent the smallest executive unit of healthcare organizations. Many interact with persons with a migrant background on a regular basis. They work on the “focal point of health literacy” [19] (p. 3) as they are expected to ensure an effective and satisfactory flow of information and to support their migrant patients in finding, understanding, appraising, and applying the information exchanged. Thus, effective oral and written communication between healthcare professionals and healthcare consumers is one of the central aspects of organizational health literacy [20]. Both patients’ health literacy limitations [21, 22] and limited language proficiency [20, 23] are common barriers to patient–provider communication. Some studies have explored the perceptions and views of migrants towards healthcare services including, for instance, satisfaction with healthcare professionals or the barriers and facilitators to healthcare services [24–26], and some have explored migrants’ views on healthcare services in relation to health literacy [27]. However, there remains little evidence on the health literacy-related challenges, needs, and applied solutions in delivering healthcare for people from diverse cultural and linguistic backgrounds from the perspectives of healthcare professionals.

### Aim

This qualitative study aimed to examine the health literacy-related challenges, needs, and applied solutions of healthcare professionals when engaging with their patients with a migrant background. Based on the integrated model of health literacy as our guiding analytic framework (see Fig. 1) [3], we examined the societal and environmental determinants, situational conditions, and personal factors, which may influence health literacy (i.e., access, understand, appraise, and apply health information) in transcultural treatment settings from the perspective of healthcare professionals in Germany.

## Methods

The present study is part of an overarching project of Gender-specific Health Literacy in Individuals with a Migration background (GLIM), including systematic reviews [28–30] and a further qualitative analysis on gender-specific aspects of health literacy, published previously [19].

### Study design

We conducted five focus group discussions with healthcare professionals (*N* = 31) between January 2018 and March 2019 in Cologne, Germany. Focus group discussions are moderated discussions in which small groups of participants are stimulated to discuss about a given topic by means of a targeted information input [31].

### Participants and recruitment

Inclusion criteria were a degree in a health-related profession, at least 2 years of work experience in a clinical role (e.g., as a nurse or physician) or in health-related counselling, regular contact with persons with a migrant background, and sufficient German language skills to participate in a discussion held in German. We applied purposive and snowball sampling to recruit healthcare professionals in the area of Cologne, a metropolitan city in West Germany, through a variety of different channels. We used a local guide for migrants that contains a list of registered physicians, therapists, clinics, counselling services, and pharmacies with diverse language competencies and clustered potential participants according to their profession and language proficiency. In addition, we placed local announcements in hospitals and distributed them by the nursing directorates, screened online search engines, and activated researchers’ professional contacts. Participants were invited via e-mail or by post.

In total, we invited 183 healthcare professionals to participate. In addition, we asked the executive staff of 38 institutions (e.g., ambulant nursing services, hospitals and joint practices) to share the information with their medical staff. Healthcare professionals who signaled a general interest in participating in one of the focus group discussions received further information about the study. To avoid uneasiness, we did not allow participants who shared a hierarchical work relation to participate in the same focus group discussions.

### Study setting and data collection

For the purpose of pretesting, we conducted two focus group discussions with *N* = 10 researchers from the department of Medical Psychology and the Cologne Center for Ethics, Rights, Economics, and Social Sciences of Health (CERES) at the University of Cologne. All had extensive experience in qualitative research methods. Six had additional practical experiences in the health system (e.g., nurse, psychologist), and six had a migrant background. The first pretest focused on methodological and ethical issues with regard to facilitating the focus group discussions (e.g., how to avoid the use of discriminatory language or how to stimulate the discussion without too much interference from the researchers). This further served to develop the semi-structured interview guide from an initial set of questions. The aim of the second pretest was to pilot and refine the interview guide, which we provide as Additional File 1. It was continuously evaluated and adapted in the course of the research process [31].

We conducted the focus group discussions at CERES. Prior to each focus group discussion, all participants received written participant information that included a brief description of the project and its aim, the conduct of the focus group discussion, and information on data security. In addition, the participants filled out a socio-demographic questionnaire including items on age, gender, migrant background, place of residence, and occupation. Three researchers (AB, DC, AA) moderated the focus group discussions; only one took on the role of the leading moderator to structure the discussion and give the participants a clear point of orientation. After the moderators were introduced, the integrated model of health literacy [3] and the definition of a migrant background were presented to the participants to provide them with an understanding of the project’s theoretical background. The moderator appealed to the participants to treat everything said during the discussion as confidential and encouraged them to elaborate on their own experiences, whether they were in line with those of the other participants or not. The audio recording began after the participants gave their informed consent (written and oral). The moderator initiated the discussions by encouraging the participants to take 3 min time to think about a concrete situation in their everyday work life that was particularly challenging with regard to health literacy and interaction with people with a migrant background. Probing questions included, for example: *“How did you deal with these challenges?”, “What would you have needed to meet this challenge?”* or *“What do you think your patient/client would have wished for in this situation?”.* All sessions lasted 120 min and ended with a reflection and a feedback round, on which basis the interview guide was evaluated and adapted. The participants received reimbursement of 25 €.

Each audio recording was transcribed verbatim in German language—the reported results in this article were translated in English using back-translation technique [32]. All cited quotes are provided in both German and English as Additional File 2. A research assistant, present in the background, wrote the minutes, and the researchers took additional field notes to document important thoughts that arose during the discussions. We report the results of this study according to the consolidated criteria for reporting qualitative studies (COREQ) [33].

### Analysis

We conducted a theory-guided qualitative content analysis according to Kuckartz (2019) [34]. The framework that guided our analysis was the integrated model of health literacy (see Fig. 1) [3]. We used a deductive–inductive categorization procedure for the analysis. This so-called abductive approach can be used to examine the implications of the applied analytical framework against the collected data and to discover meaningful patterns in, and gain a complete understanding of, the findings [35].

AB and DC deductively developed a set of categories that reflected the research question of healthcare professionals’ perceived challenges, needs, and applied solutions in communicating and interacting with their migrant patients as well as the categories drawn from the underlying framework of health literacy [36], such as the four steps of health information processing: access, understand, appraise, and apply health information. Inductive categories were derived from the data. They were subordinated to the deductive main categories or served as new main- or sub-categories, if new themes arose in the data analysis process or the data indicated the need for further distinction. AB and DC independently developed a category system alongside the transcript of the first focus group discussion. The two independent category systems were then approved, merged and converted into a common category system. On the basis of this category system, AB and DC independently coded each transcript and subsequently compared and discussed the codings. Potential discrepancies were resolved by involvement of the third author (AA). Throughout the analysis process, the category system was discussed with the author team and adapted if necessary.

We performed the coding and categorization using the MAXQDA 18.2.3 software [36], beginning with the analysis of the main categories and subsequently searching for interrelations between main- and sub-categories alongside the consented category system [37].

## Results

Thirty-six healthcare professionals expressed their interest. Three healthcare professionals did not show up and another two expressed interest but were un-able to participate at the agreed appointments. Five focus group discussions of between four to nine participants were conducted. In total, 31 healthcare professionals participated in one of the focus group discussions. One person attended upon the recommendation of a previous participant. We recruited participants until saturation was reached with regard to the categorized responses [38]. Table 1 provides an overview of the participants’ characteristics.

**Table 1:** Characteristics of focus group participants

Characteristics of focus group participants (*N* = 31)	
Factor	n
Sex	
Male	15
Female	16
Age (years)	
age range	28–71
25–34	5
35–44	11
45–54	8
55+	7
Migrant background	
Yes	16
No	15
Region^a^	
Europe	5
Turkey	6
Other (Arabian region, Africa, Asia)	5
Occupation	
Physicians/Psychologists	15
Nursing	7
Other healthcare professionals^b^	9
Setting	
Outpatient/counseling	7
Outpatient/therapeutic	18
Inpatient	6
Specialty^c^	
Psychosocial/psychiatric care	13
Medical (physical) care	22
Client base	
Adults	26
Family	5

^a^Region of origin in participants with a migrant background

^b^including e.g., ergo therapist, physio therapist, trauma counsellor or speech therapist

^c^Multiple answers were possible

The healthcare professionals did not perceive major differences between the descendants of immigrants and the German majority with regard to their health literacy. Therefore, the following results mainly reflect the participants’ views on interactions with first generation migrants with a low German language proficiency.

### Four steps of health information processing (access, understand, appraise, and apply)

We describe the results alongside the integrated model of health literacy [3], starting with the four steps of health information processing (access, understand, appraise, apply) followed by the factors that influence the entire flow of information in transcultural treatment settings (societal and environmental factors, situational factors, and personal factors). Thereby, we focus on the perceived challenges reported by the healthcare professionals on the one hand, and the already applied solutions that they perceived as effective for addressing these challenges, on the other hand. We do not report the perceived needs for addressing these challenges separately, as they were often stated implicitly in relation to the challenges and applied solutions. The deductive categories *challenges* and *applied solutions* with regard to *accessing, understanding, appraising,* and *applying* health information and the respective inductive subcategories are shown in Table 2.

**Table 2:** Categories related to the four steps of health information processing

Category^a^	Processing step^b^	Subcategory^c^
Challenges	Access	• Mismatch between provision and actual use of health services
	Understand	• Uncertainty about the causes of unsuccessful communication
	Appraise	• Insecurity in dealing with patients’ needs and expectations • Patients’ distrust in healthcare professionals and the German health system
	Apply	• Patients’ non-compliance with medical appointments
Applied Solutions	Access	• Easily accessible services and outreach counselling
	Understand	• Recourse to professional interpreters and cultural mediators • Recourse to lay interpreters (medical staff, relatives)
	Appraise	• Initiating unnecessary examinations to regain patients’ trust
	Apply	• Patience in communicating health information to patients

^a^Categories *deductively* derived from the objective of the study

^b^Subategories *deductively* derived from the guiding model (Sorensen et al) [3]

^c^Subcategories *inductively* derived from the statements of the healthcare professionals

#### Challenges and applied solutions related to *accessing* health information and services

##### Challenge: Mismatch between provision and actual use of health services

Some healthcare professionals described a mismatch between the provision and the actual use of health services and difficulties in reaching persons with a migrant background for certain measures and healthcare services. They assumed this was due to reasons including distrust in healthcare professionals and the German health system (see also challenges related to the appraisal of health information), a lack of (system-)knowledge, and a lack of involving people with a migrant background in the development of health information and the delivery of health services. One participant emphasized the latter by indicating that *“many in the* [African] *community* [are] *actually very active and* [do] *a lot of educational work* ( … ) *and I believe the* [their] *work is seen far too little.”* [HCP 12, other, outpatient, without migrant background].

Thus, many health services, even when linguistically adapted, did not meet the needs of migrants. Another participant described a counselling service for pregnant mothers and those who have recently given birth in refugee homes. However, the refugee women did not use this service. The midwives asked one of the women who was close to giving birth: “*‘why don’t you come* [use the service]?*’ And then she said, ‘I gave birth to my other four children at home with my neighbor in the kitchen, I’m glad that I’m here, that my children can play outside and I know they’ll all come back in because no bombs are falling’ and she didn’t know that there’s a maternity passport, that there are preventive examinations, that they are free of charge, the check-ups”* [HCP 6, doctor, outpatient, without migrant background].

##### Applied solution: Easily accessible services and outreach counselling

It was stressed several times that easily accessible services were perceived as helpful for reaching people with limited language skills and low (system-)knowledge for certain health services. In particular, outreach counselling, i.e., direct personal or telephone contact with persons in need of help, was rated as effective, whereas the mere provision of written information in the form of flyers or brochures was rated less useful. One participant reported that *“they* [members of the African community] *say ‘we don’t need all these flyers, we need a direct contact in order to dismantle these hurdles’”* [HCP 12, other, outpatient, without migrant background].

Another participant emphasized *“that this is an important point, because people *[with a migrant background] *don’t come to the counselling centers like that. So, outreach work, I think, is really a key.” *[HCP 2, nurse, outpatient, with migrant background].

#### Challenges and applied solutions related to *understanding* health information

##### Challenge: Uncertainty about the causes of unsuccessful communication

Many healthcare professionals stated that it was sometimes difficult to distinguish between difficulties in understanding health information due to low literacy skills or due to considerable language barriers on the part of the patients—sometimes, it was assumed to be a combination of both. This would lead to challenges in conveying information in a targeted manner and was considered to be equally stressful for both sender and receiver.

*“And if there is a language barrier in addition* (...) *then it is almost impossible to even judge it. Someone is silent and you assume that it’s because of the language, but maybe it has a completely different cause and you just don’t realize it because you can’t grasp it”* [HCP 23, nurse, inpatient, with migrant background]

##### Applied solution: Recourse to professional interpreters and cultural mediators

Nearly all healthcare professionals emphasized a need for the sufficient funding of professional interpreters or cultural mediators who do not only speak the same language as the patient but also share the same cultural norms, values or religion. Participants with access to professional interpreters from diverse cultural and linguistic backgrounds repeatedly emphasized the positive impact both on the well-being of their patients as well as on the healthcare professionals’ own workload.

*“Thank God we have the possibility to call an interpreter in our clinic and I attach great importance to the fact that the one who comes from Iran, that he gets an Iranian interpreter and not one from Afghanistan, who speaks the same language, but they do not have the same cultural background.”* [HCP 26, nurse, inpatient, w mb]

Particular emphasis was placed on the ease of use and accessibility of so-called *“video interpreters”* who offer interpreting services via video conference, provided that the costs are covered. Participants who have already had the possibility to use video interpreters in an outpatient consulting setting rated them as a helpful, quickly available, and correspondingly time-saving method to overcome language barriers as physical contact is eliminated and interpreters for various languages are made quickly available via video conference.

*“Yeah, but it works. Well, we often accompany families who don't understand anything in the clinic and where the cleaning lady or whoever tried to* [help] *or the older son or the younger daughter and we take the interpreter, the video interpreter now very often with us to the clinic* ( … ) *And we experience this as very, very helpful.*” [HCP 6, doctor, outpatient, without migrant background]

##### Applied solution: Recourse to relatives or medical staff as lay interpreters

Participants indicated on several occasions that the recourse to relatives as lay interpreters could be helpful in some situations, but could not compensate for the use of professional interpreters. Some doctors reported that they instructed their native-speaking medical staff to interpret during the treatment situation. However, some stressed that this measure could not be considered equally effective and satisfactory for all treatment situations, especially when shameful topics were discussed and the limits of confidentiality stretched. One physician summarized the problem as follows:

*“For a while, I had a female doctor's assistant who was also Turkish-speaking. It doesn't help either, because she would say: ‘No, I don't want to translate anything about this topic* [erectile problems]’. *There you are left alone”* [HCP 17, doctor, outpatient, without migrant background]

#### Challenges and applied solutions related to the *appraisal* of health information

##### Challenge: Insecurity in dealing with patients’ needs and expectations

Many healthcare professionals reported a general uncertainty in responding to the needs of migrants with low language proficiency. Thus, appraising the statements of their patients regarding their needs and expectations appeared challenging. They stressed that a mutual understanding was closely related to knowledge of cultural habits, expressions of pain and dealing with issues of shame. One healthcare professional explained that he received intercultural training but wished he had been taught more about how to sufficiently respond to his African patients’ needs: “*they communicate in English or French and that works linguistically quite well, but this is the group where I often realize that you don’t really know how they tick”* [HCP 11, doctor, outpatient, with migrant background].

This statement was strongly supported by the other participants.

*“Of course, I feel the same way, although I have the impression, especially with African or Asian patients, that I don't understand their facial expressions.* (...) *what do they mean? Yes, it's often something completely different from what I understand”* [HCP 14, doctor, outpatient, without migrant background].

##### Challenge: Patients’ distrust in healthcare professionals and the German health system

Almost all participants reported a pronounced distrust in German institutions and healthcare professionals on the part of many persons with a migrant background, which led to a negative appraisal of the information provided by the healthcare professionals. Some participants supposed that a relationship between language barriers, experiences of discrimination, but also a lack of (system-) knowledge were reasons for an increased distrust in healthcare professionals and the German health system. One participant assumed that the present differences between health systems—even within the European Union—contributed considerably to this feeling.

*“*[In Romania]*, people generally have a broad-spectrum antibiotic at home* [because these are freely available there] (...) *and then of course they come, people come here into this system and then we say: No, we don't do that, we don't give antibiotics just because you have a sore throat.* (...) *Of course, this creates a relationship of mistrust and insecurity towards our system, towards the doctors”* [HCP 25, doctor, inpatient, with migrant background]

In this regard, many participants perceived that their migrant patients often suspected they were being discriminated by German healthcare professionals.

*“Sometimes* (...) *the accusation of racism comes up, that others would certainly be treated better in the situation and would get a different* [better] *treatment.”* [HCP 24, psychologist, with migrant background]

However, this was not reported by healthcare professionals who themselves had a migrant background. These participants assumed that the health information they delivered to their migrant patients was appraised as more trustworthy than the information delivered by their German colleagues. For example, a Turkish doctor reported that he was often confronted with culture-specific ideas regarding the end of life in the intensive care unit. When it came to communicating treatment decisions not in line with the culture-specific perceptions, philosophical, or religious beliefs of the relatives of his Turkish patients, he felt he was in a more favourable position than his German colleagues:

*“Many* [people with a migrant background] *feel that they are, yes, being treated badly or that they have disadvantages due to their migrant background, that they are patronized* (...)*. I am not confronted with that. They take it from me that they say, okay, you are a doctor, a Turkish doctor and you give everything and when I then say, ‘it's good now, he won't make it, your father’. Then, they believe me more than if a German colleague would say that now.”* [HCP 30, doctor, inpatient, with migrant background]

##### Applied solution: Initiating unnecessary examinations to regain patients’ trust

Some healthcare professionals reported that they sometimes found themselves initiating unnecessary examinations to pacify conflicting situations and thereby regain trust when they felt that they were accused of disadvantaging their migrant patients over those of the German majority population. This strategy was rated as unsatisfactory, inefficient, and costly.

*“I think that probably happens quite often every day that an examination is ordered in order to, let's say, pacify the situation. Starting maybe with an ECG* [electrocardiogram] *and up to bigger things* [more expensive examinations] *and so* [you can imagine] *what that also means for the* [health] *system”* [HCP 23, nurse, inpatient, with migrant background]

#### Challenges and applied solutions related to *applying* health information

##### Challenge: Patients’ non-compliance with medical appointments

A central challenge in the outpatient care of patients with a migrant background, especially those who have recently immigrated or who grew up in countries with very different health systems, was the non-arrangement of or non-compliance with appointments: “*Somehow, making appointments doesn’t work.”* [HCP 17, doctor, outpatient, without migrant background]. One possible reason provided by the healthcare professionals was the patients’ lack of knowledge of the German health system.

##### Applied solutions: Patience in communicating health information to patients

Some healthcare professionals emphasized the importance of communication and of establishing the reasons for the patients’ non-adherence. A lack of knowledge about the health system in recently migrated persons could be met with patience and friendliness. Other immigrants, however, who have been living in Germany for many years were expected by the healthcare professionals to know the system better. These patients should be treated “*friendly but firmly,”* as one doctor of Arab descent stated. Overall, this challenge does not yet seem to have been solved satisfactorily by most of the participants.

*“*[I]*t's very difficult to teach them that it's not possible* [to treat them] *without an appointment and that they have to get an appointment and sometimes we discuss for so long* (...) *But that was in the beginning* [when patients have just immigrated], *I have to say, in the meantime it's getting much better that they have understood that. I explain it calmly and I think they learn over time.”* [HCP 13, doctor, outpatient, with migrant background]

### Factors influencing the entire flow of information in transcultural treatment settings

The participants reported that certain societal and environmental, situational, and personal factors influenced the entire flow of information (i.e., health literacy) between healthcare professionals and their migrant patients. Therefore, these could not be assigned to one of the four steps of health information processing. Table 3 shows the deductive categories *challenges* and *applied solutions* with regard to the *societal and environmental factors, the situational factors and the personal* factors that influence health literacy in transcultural treatment settings and the respective inductive subcategories.

**Table 3:** Categories related to the factors that shape health literacy in transcultural treatment settings

Category^**a**^	Factors that influence health literacy	Subcategory^**d**^
Challenges	Societal and Environmental Factors^b^ • System-related factors^c^	• Systemic lack of time and economic pressure
	Situational Factors^b^ • Psychosocial/psychiatric vs. medical (physical) care^c^ • Inpatient vs. outpatient care^c^	• Planning and controlling the current workload in outpatient care
	Personal Factor^b^ • (Shared) migrant background^c^	• Ad hoc interpreting outside one’s own treatment situation
Applied solutions	Societal and Environmental Factor^b^ • System-related factors^c^	• Investment of additional, unpaid time • Falling back on stereotypes and prejudices to save time
	Personal Factor^b^ • (Shared) migrant background^c^	• Refusal of interpreting for others or providing treatment in native language • List of staff who speak foreign languages

^a^Categories *deductively* derived from the research question

^b^Categories *deductively* derived from the guiding model [3]

^c^Categories *inductively* derived from the statements of the healthcare professionals

^d^Subcategories *inductively* derived from the statements of the healthcare professionals

#### Challenges and applied solutions related to *societal and environmental* factors

##### Challenge: Systemic lack of time and economic pressure

The participants reported that, in the context of migration, known issues related to the delivery of healthcare for the majority population appear to be intensified. Above all, system-related factors such as a systemic lack of time and economic pressure were mentioned as aggravating the effective flow of information between healthcare professionals and the patients who required special attention in treatment settings. The participants emphasized an omnipresent lack of time as being present in both the outpatient and the inpatient sectors.

*“And in the entire health system, I believe that what I have learned in the last 30 years, nothing or everything works so badly because we have too little time.”* [HCP 9, other, outpatient, without migrant background]

Some participants explicitly named the German accounting system, which is based on lump-sum fees in the outpatient sector and Diagnosis-Related Groups (DRGs) in the inpatient sector, as reason for high economic pressure. The accounting system was regarded as particularly disadvantageous for healthcare professionals who dealt with many migrant patients who required special attention and time (e.g., due to language barriers).

*“I think that a very big problem is that there are lump-sum fees* [in the German health system]*. There is indeed the depressed woman who comes* [who] *is of German origin and* (...) [on the other hand] *a person with a migrant background who does not understand the language* (...) *you have to invest more time. Maybe you have to invest more money, and in the end, you don't get paid for it.”* [HCP 27, nurse, inpatient, with migrant background]

##### Applied solution: Investment of additional, unpaid time

The most frequently applied solution to tackle the problem of time pressure was the investment of extra time beyond systemic guidelines. For example, some participants reported that they would often invest time beyond their actual capacities to meet the needs of patients with a migrant background, but this was at the expense of their personal free time.

*“And then I also take a lot of time and often it's the underlying conditions* [of the health system] *that make it difficult, so mostly it's personal free time that I take”* [HCP 14, doctor, outpatient, without migrant background]

Another outpatient doctor reported that he often invests additional time for his patients who require more support, but reduces treatment time for others whenever possible.

*“That’s the way it is in a general practice, you have to take time away from one to have time with the other. But if you then have someone with a migrant background, where you notice that it doesn't work linguistically, then *[more time is needed, but] *I can't kick them out. They haven't done anything [wrong].”* [HCP 19, doctor, outpatient, without migrant background]

##### Applied solution: Falling back on stereotypes and prejudices to save time

Some of the participants reported that they caught themselves and their colleagues falling back on stereotypes and prejudices to avoid insecurity and to save time when work was particularly hectic. For example, some health care professionals described the so-called “morbus mediterraneus”, a stereotype that labelled people from Southern European countries as being particularly plaintive persons who expressed physical pain intensely. Therefore, the patients’ pain intensity would have been doubted by some healthcare professionals. The participants reported that some healthcare professionals tended to resort to this cognitive short-cut to save time. These stereotypical ascriptions would, in the worst case, lead to poor health care for people with a southern European migrant background because their symptoms were not taken seriously.

*“Well, I have now clearly noticed in our everyday life that due to this time pressure, due to this stress and the fact that we get our patients through quite quickly, we often resort to prejudices and stereotypes and then one simply says: ‘Okay, yes, Mediterranean patient, just morbus mediterraneus, let's just do analgesia and send them back home.’”* [HCP 25, doctor, inpatient, with migrant background]

#### Challenge related to situational factors

##### Planning and controlling the current workload in outpatient care

Situational factors such as the treatment setting (inpatient versus outpatient care) were reported to generally influence the flow of information in transcultural encounters. Some challenges, however, were reported to be aggravated in the context of migration. For example, planning and controlling the current workload in the outpatient care (see also challenge ‘Patients’ non-compliance with medical appointments’) was stated as a problem. In this regard, the most significant differences were perceived between psychiatric and medical (physical) outpatient care. In Germany, for both the outpatient, psychiatric and the medical (physical) care, patients have to make appointments, but people with acute health problems will still be treated. This is every-day practice in medical (physical) care, but less so in psychiatric care. A physician reported on his young migrant patient who presented to the practice without an appointment for his non-acute problem. Like many other healthcare professionals working in the outpatient setting, he referred to the lack of time to discuss such problems in detail:

*“But you* *shouldn't forget that a doctor's practice also means an average of five minutes of medicine. So now I can't sit down with a young man who presents this problem* [erectile dysfunction], *and say, now I take half an hour of time and listen exactly where the problem is. Then the waiting room would overflow.”* [HCP 17, doctor, outpatient, without migrant background]

Another participant concurred with this: *“So, yes, so I thought, there is certainly a difference. I have a purely appointment-based practice, an order-based practice as a psychotherapist, and so I don’t have the problem that patients just come along, yes. That happens to me much less.”* [HCP 16, psychologist, with migrant background].

Challenges and applied solutions related to the personal factor ‘(shared) migrant background’

More than half of the participants were first- or second-generation migrants themselves. The majority of these healthcare professionals had roots in Turkey (see Table 1). Participants who had a personal migrant background perceived that the delivery of healthcare for migrant patients was full of opportunities with regard to reducing distrust against healthcare professionals (see challenges related to the appraisal of health information). However, they also reported challenges, particularly with regard to using their language competencies in everyday work.

##### Challenge: Ad hoc interpreting outside one’s own treatment situation

Participants reported mixed feelings about using the shared language and culture. On the one hand, they described situations in which they actively decided to translate for German colleagues or to provide language-concordant treatment. Such situations were perceived as enriching. On the other hand, this was not the case when they were obliged to interpret for others or were *“caught off guard”* by requests at short notice. Interpreting was sometimes perceived as *“stressful”* due to the high workload across all healthcare settings. Some reported that they perceived the “*high responsibility”* associated with lay interpreting as a burden.

*“I mean, I come from a region where people don't speak pure Turkish. That's quite a slang* ( … ). *At some point I didn't want to do that anymore and I found it rather burdening and things often happened between door and hinge, and I didn't want to be held responsible for it.”* [HCP 28, nurse, inpatient, with migrant background]

##### Applied solution: Refusal of interpreting for others or providing treatment in native language

Strategies to escape interpretation included the denial of ad hoc interpreting in certain situations or even the denial of interpreting in general. One participant reported that he sometimes even covered his name to hide his migrant background or that he pretended he couldn’t speak Turkish. Another participant agreed: *“I have many Turkish colleagues who handle this similarly and don’t speak Turkish at all and say, okay, you* [the patient] *are in a German hospital, then you have to sort it out in German somehow”* [HCP 30, doctor, inpatient, with migrant background].

##### Applied solution: List of staff who speak foreign languages

Several participants reported that their organizations had lists of staff who speak certain languages to enable other healthcare professionals to quickly reach out for them in ad-hoc situations. This was perceived as well received, as these lists were developed on a voluntary basis. However, the quality of interpreting was described as being person-dependent.

*“We have, for example, a list of foreign languages spoken by the staff that was known in the clinic. Basically, every nation was represented ( … ) Yes, the quality was always very different, depending on who* [of the staff] *actually came to translate.”* [HCP 27, nurse, inpatient, with migrant background]

## Discussion

The aim of this study was to investigate how healthcare professionals perceive the health literacy-related challenges, needs, and applied solutions in transcultural interactions. We explored the societal and environmental, situational, and personal factors that potentially shape health literacy (i.e., access, understand, appraise, and apply health information) in transcultural treatment settings from the perspective of those providing treatment.

We asked the participants for concrete situations that they found challenging in their interactions with persons with a migrant background. In this regard, we referred to them as either first- or second-generation migrants. Interestingly, the participants related almost all statements to first-generation migrants and repeatedly emphasized that second-generation migrants who had grown up in Germany and were highly acculturated (e.g. in terms of language and social membership) had health literacy-related needs similar to those of the German majority population. In line with this, other studies have also indicated that having a migrant background does not, per se, imply a lower level of health literacy—at least among young people when compared to their peers without a migrant background [39–41]. It might, rather, function as a multiplier in reproducing health-related inequalities [29], as health literacy has a social gradient [5], and differences in health literacy levels can also be explained by an average lower level of education or lower social status rather than by the migrant background itself [42].

Many statements were related to people who migrated from very different health systems to Germany, often referred to as first-generation migrants of Turkish or Arab descent. This may be because some participants themselves had a Turkish (*n* = 6) or an Arab migrant background (*n* = 3), thereby attracting more patients of the same origin. Another possible reason is that Turks and Arabs belong to the largest immigrant groups in Germany [43] and are, therefore, very present in the healthcare sector.

**Fig. 2:**
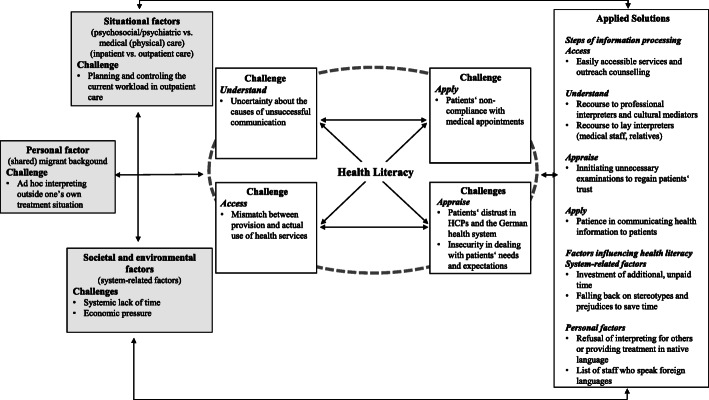
Pathways of deductive main categories **(bold)** and exemplary inductive subcategories of health literacy-related challenges and applied solutions in the transcultural treatment settings. HCPs: healthcare professionals

The results of this study indicate that a successful interaction in transcultural treatment situations is not exclusively a question of the individual knowledge, motivation, skills, and abilities of the healthcare professionals and their patients. Rather, health literacy in transcultural treatment settings appears to be an interplay of several external and internal factors that influence information delivery on the one hand and information processing on the other. This finding is in line with other qualitative studies that emphasize the social-relational character of health literacy, discussing it as social practice [44] and communicative action [45]. For the purpose of this study, the integrated model of health literacy [3] provided helpful guidance. However, in the context of transcultural treatment settings, we found the four steps of health information processing—access, understand, appraise and apply—to be neither distinctive nor consecutive. Instead, our results suggest that they are interactive, thereby reinforcing each other, and are influenced (to varying degrees) by individual, situational, societal and environmental factors. In addition, we found the personal, situational, and environmental factors that influence health literacy to be strongly interrelated. They had an impact on the kind and expression of challenges perceived, but also on the choice of and satisfaction with the applied solutions, which, in turn, influence the development of current and future patient-provider relationships. Therefore, not all inductive subcategories were subordinated to one of the a priori set distinct deductive categories. In addition, we did not report perceived needs separately, as they were often implicitly stated in relation to a challenge or an applied solution. For example, the participants reported a need for professional (culture-concordant) interpreters. Likewise, those who already had access to professional interpreters reported this as a helpful solution. Thus, the reported results reflect those categories that inductively emerged from the data and that most closely matched the respective deductive main category. Figure 2 shows the pathways and interrelations of the deductive **(bold)** and inductive categories related to the four steps of health information processing described in the results.

One of the major challenges related to accessing health information and health services was a mismatch between the provision and actual use of health services. Some of these services, even when linguistically adapted, do not seem to meet the needs of persons with a migrant background. Accordingly, the involvement of migrants (i.e., people concerned) in the development and implementation of such services, their easy accessibility, and outreach counselling were rated as helpful solutions to this challenge. The European study RESTORE, for instance, found positive effects on knowledge, skills, and clinical routines when they involved migrants along with other stakeholders in the implementation of guidelines and training initiatives on cross-cultural communication [46].

Our results indicate that in transcultural treatment settings, the respective parties’ understanding of each other depends on system-related factors such as time and the accessibility and funding of professional interpreters. Some participants reported that it was sometimes difficult to distinguish language-related problems in understanding health information from literacy-related difficulties in understanding medical information in general. Communicating personal health issues can be highly challenging, even when people are literate in their native language. Language barriers and culture-specific differences in the understanding of illness or the expression of pain can be additionally challenging for persons from diverse migrant backgrounds. This can, in turn, lead to misunderstandings and false conclusions being made about the person’s health literacy. Thus, the participants regarded the funding of cultural mediators or professional interpreters with at least some professional medical knowledge (e.g., culture concordant video interpreters) as significant for overcoming the major language-related challenges in the provision of care. This finding is supported by other studies that include the perspectives of either health professions [47] or migrant patients [48]. For instance, one study conducted in Switzerland found that two thirds of the participating physicians who face language barriers have never had access to a professional interpreter, even though 87.8% would appreciate their presence in clinical practice [49]. However, our findings indicate that the mere provision of translated information material and interpreters, though indispensable, cannot solve the deeper challenges present in the provision of healthcare for people of diverse migrant backgrounds. Unless health literacy, cultural particularities and language barriers are not addressed simultaneously [50], the mutual satisfactory flow of information remains a fervent wish rather than clinical reality in transcultural treatment situations.

A key component for a mutually satisfying flow of information was trust. Our findings reveal that this factor is influential, particularly with regard to accessing health information and services as well as appraising the health information exchanged (see Fig. 2). This finding is supported by other studies which indicate that distrust in health services is one of the major barriers to healthcare access [24], whereas patients’ trust in healthcare professionals can promote a willingness to seek advice, the acceptance of medical recommendations, improved treatment adherence and satisfaction, and subjective health outcomes [51]. Using heuristic shortcuts (e.g., stereotypes) when people have to process complex information under time pressure, is a well-known psychological phenomenon [52]. For example, some healthcare professionals described the stereotypical ascription “morbus meditarreneus” as being used to label the allegedly exaggerated expression of pain by persons from Southern European countries. Such stereotypes may serve the maintenance of subtle racism against people with a migrant background, which is still present in health systems [53, 54]. Furthermore, it can lead to even more distrust on the part of the patients [53] and, in the worst case, to poor medical decisions that disadvantage minority patients [54]. This is because trust is the result of people’s lived experiences, and it shapes how future experiences are perceived. It can be described as a *“forward-looking evaluation of an ongoing relationship”* [55] (p. 617). Thus, trust is determined to a considerable extent by the experiences and expectations of both the healthcare professionals and their patients. Both parties’ efforts to create trustful relationships may, therefore, improve the flow of information. This could be supported by (1) the implementation of culture-sensitive measures such as sufficient funding of cultural mediators and professional interpreters, (2) the integration of intercultural learning contents into the educational curricula for medical professions, and (3) the involvement of people with a migrant background in the implementation and development of health information and services.

Time and resource constraints are well known issues in many health systems worldwide [51]. Accordingly, of all the factors that influence health literacy in transcultural treatment settings, the healthcare professionals perceived system-related factors, such as the systemic lack of time and economic pressure as impeding the flow of information in transcultural treatment settings the most. The participants perceived these conditions as highly stressful. In the context of migration, however, these known issues of the German health system were perceived as particularly aggravating. The healthcare professionals reported that they required extra time and effort to treat patients with low language proficiency due to language barriers, a lack of (system-)knowledge, or low literacy skills on the patients’ side. The most frequently mentioned strategy to manage these challenges included the investment of additional, unpaid time. This strategy was not always successful but it was seen as the most effective method for solving these issues. The issue of time pressure was reported mainly by participants working in the medical (physical) care, whereas the psychiatric professions seem to be less affected in this regard. This result reflects the importance of considering the particularities of different treatment settings when pinpointing specific challenges in transcultural interactions.

More than half of the healthcare professionals had a migrant background themselves. These healthcare professionals found that this personal factor positively influenced the establishment of trustful relationships and the acceptance of treatment recommendation. In line with this, a recent study from Germany found that a shared migrant background improved trust in the physician, reduced reactance-related outcomes, and improved prevention-related knowledge transfer in patients with a Turkish migrant background, especially in those with low health literacy [56]. However, challenges were also reported. In particular, the controversial discussion regarding interpreting outside one’s own treatment situation revealed that it should not be assumed that a healthcare professional with certain language skills is willing to or feels secure to use these skills in a professional context. Some healthcare professionals who reported frequent ad hoc interpreting outside their own treatment situation referred to two particular issues: firstly, an additional increased workload during the time of interpreting, and secondly, a fear of not translating properly in the short time available. The reported concerns of one nurse that he may be translating incorrectly because his own language skills may not be sufficient to translate complex medical issues *“between door and hinge”* [HCP 26, nurse, inpatient, with migrant background] is in line with empirical evidence indicating that nurses untrained in interpreting frequently make mistakes when translating for other healthcare professionals. This may have considerable negative clinical implications for the patients affected [57]. In particular, ad hoc interpreting may result in incorrect medical interpretation [58]. Therefore, the overriding majority of professional associations of interpreters, training institutions, and scientists demand professional interpreting in healthcare and advise against non-professional solutions [59].

### Strengths and limitations

To the best of our knowledge, this was the first study that aimed to investigate the health literacy-related challenges, needs, and applied solutions from the perspective of healthcare professionals and systematically analyze the personal, situational, and environmental factors that shape health literacy in transcultural treatment settings by applying an established health literacy framework [3].

Further strengths of this study are that the research was conducted by an interdisciplinary and multicultural research team. This meant the research team involved an advantageous combination of different genders, expertise, views, backgrounds and focal points. Reflexivity was something we considered throughout the entire research process, beginning with the conceptualization of the focus group discussions up to the final data analysis and derivation of implications. In our research team, we repeatedly discussed our understanding of health literacy as a social-relational construct, our conception of migration, and the potential influence of our attitudes and preconceptions on the dynamic of the focus group discussions, the results, and the data analysis. For example, despite the emphasis that all opinions were welcome, an egalitarian attitude may have induced rather cautious statements to avoid prejudices and stereotypes.

One limitation of this study is that a selection bias might have occurred, as many participants were highly interested in the issue. On the other hand, some focus groups also included the researcher’s distant professional contacts. This may have contributed to a balance in the sample as they may have participated because of the researchers’ personal approach rather than their intrinsic interest in the topic. In addition, here was a slight surplus of participants working in the outpatient setting and not all focus group discussions were balanced with regard to the participants’ gender and migrant background. However, the individual focus group discussions were rather homogenous in terms of the participants’ occupation (e.g., doctors or nurses), their status and outpatient or inpatient contact with persons with a migrant background. In addition, it can be assumed that all have worked in the inpatient sector at some point, either during and after their vocational or specialist training, respectively. Thus, the reported experiences were fed to some extent from both areas. Finally, although saturation was reached with regard to categorized responses, due to the nature of qualitative research, new categories might have emerged had the participants been interviewed at another time [60].

## Conclusion

Health literacy in transcultural treatment settings is an interplay of environmental (i.e., system-related), situational and personal factors. Known issues observed in the delivery of healthcare for the majority population (i.e., systemic lack of time and economic pressure) appear to be intensified in the context of migration. These factors impede the flow of information in all treatment settings to various degrees. An increasingly diverse patient clientele indicates the growing need for culture-sensitive, health-literate healthcare organizations. A corresponding diversity in the health workforce in terms of culture, language, and gender is therefore highly desirable and should be strengthened by national finance and educational programs. Ad hoc solutions, such as recourse to healthcare professionals with a migrant background as lay interpreters, should not become the means of choice for compensating for deficits in the funding of professional interpreters. Interpreting for colleagues should be treated as an additional part of these healthcare professionals’ work and not as an additional requirement during their leisure time. Healthcare professionals who interpret for colleagues should have the choice to do so voluntarily and should be provided with the necessary time for it. Further studies in different countries with diverse health and health insurance systems are needed in order to develop and implement appropriate interventions for improving health literacy at the individual and organizational levels. These studies should involve both healthcare professionals and people with a migrant background to ensure equitable healthcare that meets the needs of all persons being involved in the treatment situation. We call upon political decision-makers to further expand the funds for interpreting services and enable such services to be used at the lowest possible threshold, for example via video conference.

## Supplementary Information

**Additional file 1.**

**Additional file 2.**

## Data Availability

Due to the qualitative nature of the project, the dataset is not publicly available. Personal narratives could be more readily associated with individual respondents. Data may be available on reasonable request. Contact person is Annika Baumeister: annika.baumeister@uk-koeln.de

## Abbreviation

HCPs: Healthcare professionals
