# Supplementary material for: “The system has to be health literate, too” - perspectives among healthcare professionals on health literacy in transcultural treatment settings
Source: BMC Health Serv Res. 2021 Jul 21;21:716. doi: 10.1186/s12913-021-06614-x (PMC8293586; doi:10.1186/s12913-021-06614-x)
Supplement: Supplementary file 1 — Additional file 1. [file 12913_2021_6614_MOESM1_ESM.docx]

**Interview guide**

**1 Greeting**

- 1. Introduction of the researchers*(professional background, personal motivation for the study) and the study’s aim
  2. Formalities (ground rules for the focus group discussion, obtaining oral consent (in addition to written consent) and starting of the audio recording)
  3. Introduction round of the participants

*Please introduce yourself briefly. What is your name, in what professional context do you work with people from a migrant background and what motivated you to participate in this study?*

**2 Stimuli**

- 1. Introduction of the phenomena of interest (health literacy, migrant background, gender)

**3 Focus Group Discussion**

**3.1 Topic I – Challenges**

**Initial question:** *Well, you all work in a health care context with women and men who have a migrant background. Please take three minutes time to remember concrete situations from your day-to-day work, for example a treatment situation with the persons themselves or with their relatives, that was very typical or maybe even special and which you still have vivid memories of - regardless of whether it was solved satisfactorily. You are also welcome to take notes on this.*

**3.2 Topic II – Applied Solutions**

*Now you have talked in detail about challenges from everyday life - your own challenges and those of the people you work with...*

- *How did you deal with these challenges?”*
- *Have you or the others in the round ever solved a similar situation satisfactorily for both sides?*

**3.3 Topic III – Needs**

- *How satisfied were you with the outcome of the situation?*
- *What did you miss in this situation*
- *What would you have needed to meet the challenge?*
- *What do you think your patient/client would have wished for in this situation?*
- *In your opinion, what were the reasons that led to this result, which you felt was good/bad?*

**3.4 Topic IV – Health literacy**

**Access**

*The people you deal with, how do they find you?*

*What do people know about health or the specific problem they come to you with?*

*How do they inform themselves about health matters?*

*What do they want to know from you, when do you think they prefer other sources of information?*

**Understand**

*How do you actively support your clients to understand and appraise health information?*

**Appraise**

*What are the reasons why certain measures or recommendations are not/highly accepted?*

*According to which criteria do you think your patients/clients assess health information?*

*How do you support them to assess which information is accurate and which information they can trust?*

**Apply**

*What obstacles, but also what factors promoting the step from knowledge to action do you identify in your professional practice?*

*How do you support your clients/patients in using the information you provide?*

*What do you think influences people with a migrant background in their decision to translate health-related information into active action?*

**Gender**

- *Imagine that the patient/client was male/female - do you think the situation would have been different?*
- *What situations have you experienced in dealing with female and male migrants in which it played a role that you yourself are a man/woman?*
- *Are there approaches specific for women or men with a migrant background for assessing which sources of health information are particularly credible?*

**Migration**

- *What differences do you observe between the different waves of migration ("guest workers", politically/religiously persecuted, flight from sexualised violence, war refugees)?*
- *Which specific challenges do you observe for the first generation (i.e. the people who migrated themselves) and which ones rather for the following generation?*

**Cultural differences/specifics**

*What difference would it have made if the person in your described had been a person without a migrant background?*

**Steering questions**

- *Does anyone else in this group have experiences regarding this kind of situation when treating male/female patients or do you remember situations which are totally different from this experience?*

When language barriers are too prominent: *Apart from language barriers, what concrete difficulties do you observe for your patients/clients in terms of information processing? To what extent could other aspects that can influence the individual as a man or woman in their attitudes towards health and illness also play a role?*

**3.7 Final questions**

- *We would now like to conclude the focus group discussion by asking you to give your brief summary of today's discussion one after another: What have you learned from this discussion round? Have we addressed all the points that are important to you? Are there any relevant aspects that you missed and that we should take up in the next focus group discussions?*
