# Supplementary material for: “The system has to be health literate, too” - perspectives among healthcare professionals on health literacy in transcultural treatment settings
Source: BMC Health Serv Res. 2021 Jul 21;21:716. doi: 10.1186/s12913-021-06614-x (PMC8293586; doi:10.1186/s12913-021-06614-x)
Supplement: Supplementary file 2 — Additional file 2. [file 12913_2021_6614_MOESM2_ESM.docx]

Appendix A – German quotes and English translation

| **Quote** | **German** | **English translation** | **Participant** |
| --- | --- | --- | --- |
| **Challenges and applied solutions related to accessing health information and services** | | | |
| **Challenge ‘Mismatch between provision and actual use of health services’** | | | |
| 1 | *„Viele aus der* [Afrikanischen] *Community* [sind] *eigentlich sehr viel aktiv und* [machen] *Aufklärungsarbeit* (…) *und ich glaube, da wird die* [ihre] *Arbeit viel zu wenig gesehen.“* | *"Many in the* [African] *community* [are] *actually very active and* [do] *a lot of educational work (..) and I believe that the* [their] *work is seen far too little.”* | HCP 12, other, outpatient, without migrant background |
| 2 | *„‚warum kommen Sie nicht?‘ Und dann hat die gesagt, ‚ich habe meine anderen vier Kinder zu Hause mit meiner Nachbarin in der Küche bekommen, ich bin froh, dass ich hier bin, dass meine Kinder draußen spielen können und ich weiß, die kommen alle wieder rein, weil keine Bomben fallen‘ und die kannte das nicht, dass es einen Mutterpass gibt, dass es Vorsorgeuntersuchungen gibt, dass die kostenlos sind“.* | *“’why don't you come [use the service]?’ And then she said, ‘I gave birth to my other four children at home with my neighbor in the kitchen, I'm glad that I'm here, that my children can play outside and I know they'll all come back in because no bombs are falling’ and she didn't know that there's a maternity passport, that there are preventive examinations, that they are free of charge, the check-ups’”* | [HCP 6, doctor, outpatient, without migrant background] |
| **Applied solution ‘Easily accessible services and outreach counselling’** | | | |
|  | *“die [Mitglieder der Afrikanischen Gemeinschaft] sagen ‚wir brauchen nicht diese ganzen Flyer, wir brauchen einen direkten Kontakt, um auch diese Hürden abzubauen‘“* | “they [members of the African community] say ‘we *don't need all these flyers, we need a direct contact in order to dismantle these hurdles’”* | HCP 12, other, outpatient, without migrant background |
| *3* | *„dass das ein wichtiger Punkt ist, weil die Leute* [mit Migrationshintergrund] ja *doch nicht so dann kommen in die Beratungsstellen. Also aufsuchend, finde ich, ist wirklich ein Schlüssel.“* | *“that this is an important point, because people* [with a migrant background] *don't come to the counselling centers like that. So, outreach work, I think, is really a key.”* | [HCP 2, nurse, outpatient, with migrant background] |
| **Challenges and applied solutions related to understanding health information** | | | |
| **Challenge: Uncertainty about the causes of unsuccessful communication’** | | | |
| 4 | *“Und wenn dann noch eine Sprachbarriere dazukommt, (…) dann kann man es ja fast noch nicht mal beurteilen. Also da schweigt jemand und ähm schweigt und man geht davon aus, dass es an der Sprache hängt, aber vielleicht hat das eine ganz andere Ursache und man kriegt das einfach nicht mit, weil man es nicht erfassen kann.”* | “*And if there is a language barrier in addition, (...) then it is almost impossible to even judge it. Someone is silent and you assume that it's because of the language, but maybe it has a completely different cause and you just don't realize it because you can't grasp it.”* | HCP 23, nurse, inpatient, with migrant background |
| **Applied solution: ‘Recourse to professional interpreters and cultural mediators’** | | | |
| 5 | *“Wir haben Gott sei Dank bei uns in* [der] *Klinik die Möglichkeit, einen Sprachmittler zu bestellen und ich lege sehr Wert drauf, dass derjenige, der aus Iran kommt, dass er ein iranischer Dolmetscher bekommt und keine aus Afghanistan, die gleiche Sprache spricht, aber die nicht die gleiche kulturellen Hintergründe haben.”* | *“Thank God we have the possibility to call an interpreter in* our *clinic and I attach great importance to the fact that the one who comes from Iran, that he gets an Iranian interpreter and none from Afghanistan, who speaks the same language, but they do not have the same cultural background.”* | HCP 26, nurse, inpatient, with migrant background |
| 6 | *“Ja, aber es funktioniert. Also wir gehen ganz häufig mit Familien, die in der Klinik nichts verstehen und wo auch dann die Putzfrau versucht hat* [zu helfen] *oder wer auch immer, oder der ältere Sohn oder die jüngere Tochter und wir nehmen den Dolmetscher, also den Videodolmetscher jetzt ganz oft mit in die Klinik (…) Und das erleben wir als sehr, sehr hilfreich.“* | *"Yeah, but it works. Well, we often accompany families who don't understand anything in the clinic and where the cleaning lady or whoever tried to* [help] *or the older son or the younger daughter and we take the interpreter, the video interpreter now very often with us to the clinic (…) And we experience this as very, very helpful.”* | HCP 6, doctor, outpatient, without migrant background |
| **Applied solution ‘Recourse to relatives or medical staff as lay interpreters’** | | | |
| 7 | *“Ich hatte mal eine Zeit lang eine Arzthelferin gehabt, die auch türkischsprachig war. Nützt auch nichts, weil die sagt dann: Nein, also bei dem Thema* [Erektionsprobleme] *möchte ich nicht irgendwie übersetzen. Da ist man allein gelassen.”* | *"For a while, I had a female doctor's assistant who was also Turkish-speaking. It doesn't help either, because she would say: ‘No, I don't want to translate anything about this topic* [erectile problems]’. *There you are left alone."* | HCP 17, doctor, outpatient, without migrant background |
| **Challenges and applied solutions related to the appraisal of health information** | | | |
| **Challenge ‘Insecurity in dealing with patients’ needs and expectations’** | | | |
| 9 | “*Die kommunizieren dann auf Englisch oder Französisch und ähm das funktioniert sprachlich so ganz gut, aber das ist die Gruppe, wo ich eigentlich oft merke, da weiß man nicht so wirklich, wie die ticken.”* | “*They communicate in English or French and um that works linguistically quite well, but this is the group where I often realize that you don't really know how they tick.”* | HCP 11, doctor, outpatient, with mb |
| 7 | *„Ganz klar, geht mir ganz genauso, wobei ich speziell bei afrikanischen oder asiatischen Patientinnen den Eindruck habe, ich verstehe nicht ihre Mimik.* (…) *ja, was meinen die? Ja, das ist oft was ganz anderes als ich verstehe“* | *“Of course, I feel the same way, although I have the impression, especially with African or Asian patients, that I don't understand their facial expressions.* (...) *what do they mean? Yes, it's often something completely different from what I understand”* | HCP 14, doctor, outpatient, without migrant background |
| **Challenge ‘Patients’ distrust in healthcare professionals and the German health system’** | | | |
| 8 | *“Man hat auch* [in Rumänien] *per se grundsätzlich ein Breitbandantibiotikum zu Hause* [weil sie dort frei verkäuflich sind (…) *und dann kommen die natürlich, kommen Menschen hier in dieses System und dann sagen wir: Nö, machen wir nicht, wir geben kein Antibiotikum, nur weil Sie gerade Halsschmerzen haben.* (…) *Natürlich entsteht dann halt so ein Misstrauensverhältnis und eine Verunsicherung gegenüber halt unserem System, den Ärzten.“* | *"*[In Romania], *people generally have a broad-spectrum antibiotic at home* [because these are freely available there] (...) *and then of course they come, people come here into this system and then we say: No, we don't do that, we don't give antibiotics just because you have a sore throat.* (...) *Of course, this creates a relationship of mistrust and insecurity towards our system, towards the doctors“* | [HCP 25, doctor, inpatient, with migrant background] |
| 9 | *„Da kommt auch manchmal der Rassismusvorwurf hoch, andere würden sicherlich in der Situation besser behandelt und würden eine andere* [bessere] *Behandlung kriegen“* | *“Sometimes the accusation of racism comes up, that others would certainly be treated better in the situation and would get a different* [better] *treatment”* | [HCP 24, psychotherapist, with migrant background] |
| 10 | *“Viele* [Personen mit Migrationshintergrund] *haben so das Gefühl, dass sie, ja, schlecht behandelt werden beziehungsweise halt durch ihren Migrationshintergrund auch Nachteile haben, bevormundet werden* (...). *Damit werde ich halt nicht konfrontiert. Das nehmen die mir halt schon ab, dass die sagen, okay, du bist Arzt, türkischer Arzt und du gibst halt alles und wenn ich dann sage ‚es ist gut jetzt, er wird es nicht schaffen, Ihr Vater‘. Dann glauben die mir eher als wenn das jetzt ein deutsche Kollege sagen würde.”* | *"Many* [people with a migrant background] *have the feeling that they are, yes, treated badly or have disadvantages due to their migrant background, that they are patronized* (...)*. I am not confronted with that. They take it from me that they say, okay, you're a doctor, a Turkish doctor, and you give everything and when I then say, ‘it's good now, he won't make it, your father‘. Then, they believe me more than if a German colleague would say that now.”* | HCP 30, doctor, inpatient, with migrant background |
| **Applied solution ‘Initiating unnecessary examinations to regain patients’ trust’** | | | |
| 11 | *„ich denke ja, das passiert wahrscheinlich täglich ganz oft, dass eine Untersuchung veranlasst wird, um ich sage mal die Situation zu befrieden, angefangen vielleicht bei einem EKG* [Elektrokardiogramm]*, und bis hin zu größeren Sachen* [teurere Untersuchungen] *und also* [kann man sich vorstellen] *was das auch natürlich für das System bedeutet“* | *“I think that probably happens quite often every day that an examination is ordered in order to, let's say, pacify the situation. Starting maybe with an ECG* [electrocardiogram] *and up to bigger things* [more expensive examinations] *and so* [you can imagine] *what that also means for the system”* | [HCP 23, nurse, inpatient, with migrant background] |
| **Challenges and applied solutions related to applying health information** | | | |
| **Challenge ‘Patients’ non-compliance with medical appointments’** | | | |
|  | *„Termine machen funktioniert irgendwie nicht“* | *“Somehow, making appointments doesn't work.”* | [HCP 17, doctor, outpatient, without migrant background]. |
| **Applied solution ‘Patience in communicating health information to patients’** | | | |
| 9 | *„*[D]*as ist sehr schwer dann beizubringen, dass das* [die Behandlung] *ohne Termin nicht geht und dann müssen sie einen Termin bekommen und manchmal diskutieren wir so lange (...). Aber das war am Anfang* [als die Patienten gerade erst immigriert sind], *muss ich sagen, mittlerweile wird viel besser, dass die das mal verstanden haben. Also ich erkläre in aller Ruhe und ich glaube, sie lernen also da mit der Zeit.“* | *“*[I]*t's very difficult to teach them that it's not possible* [to treat them] *without an appointment and then they have to get an appointment and sometimes we discuss for so long* (...) *But that was in the beginning* [when patients have just immigrated], *I have to say, in the meantime it's getting much better that they have understood that. I explain it calmly and I think they learn over time.”* | HCP 13, doctor, outpatient, with migrant background |
| **Challenges and applied solutions related to societal and environmental factors** | | | |
| **Systemic factors: Challenge ‘Systemic lack of time and economic pressure’** | | | |
| 10 | *„Und im gesamten Gesundheitswesen, das ist, glaube ich, das, was ich gelernt habe in den letzten 30 Jahren, klappt überhaupt nichts oder alles so schlecht, weil wir zu wenig Zeit haben.“* | *“And in the entire health system, I believe that what I have learned in the last 30 years, nothing or everything works so badly because we have too little time.”* | [HCP 9, other, outpatient, without migrant background] |
| 11 | *„Ich glaube, dass ein ganz großes Problem ist, dass es Pauschalen gibt* [im deutschen Gesundheitssystem]. *Es gibt tatsächlich die depressive Frau, die kommt,* [die] *deutscher Herkunft ist und* [auf der anderen Seite] *ein Mensch mit einem Migrationshintergrund, der die Sprache nicht versteht* (..) *Man muss da mehr Zeit investieren. Man muss vielleicht mehr Geld investieren und das kriegt man letztendlich nicht bezahlt“* | *“I think that a very big problem is that there are lump-sum fees* [in the German health system]. *There is indeed the depressed woman who comes* [who] *is of German origin and* (...) [on the other hand] *a person with a migrant background who does not understand the language* (...) *you have to invest more time. Maybe you have to invest more money, and in the end, you don't get paid for it.”* | HCP 27, nurse, inpatient, with migrant background |
| **Applied solution ‘Investment of additional, unpaid time’** | | | |
| 12 | *„Und dann nehme ich mir zwar auch viel Zeit und oft sind es dann eben auch die Rahmenbedingungen, die es schwer machen, also meistens ist es dann persönliche Freizeit, die ich mir nehme“* | *"And then I also take a lot of time and often it's the underlying conditions [of the health system] that make it difficult, so mostly it's personal free time that I take”* | HCP 14, doctor, outpatient, without migrant background |
| 13 | *„So ist es in einer Hausarztpraxis, Sie müssen Zeit bei dem einen rausholen, um beidem anderen Zeit zu haben. Wenn Sie dann aber so jemanden haben mit Migrationshintergrund, wo Sie merken, dass das sprachlich nicht geht, dann* [wird mehr Zeit benötigt] *Ich kann die* [aber] *schlecht rauswerfen. Sie haben doch gar nichts gemacht.“* | *“That's the way it is in a general practice, you have to take time away from one to have time with the other. But if you then have someone with a migrant background, where you notice that it doesn't work linguistically, then* [more time is needed, but] *I can't kick them out. They haven't done anything* [wrong]*.”* | HCP 19, doctor, outpatient, without migrant background |
| **Applied solution ‘Falling back on stereotypes and prejudices to save time’** | | | |
| 8 | *„Also ich habe jetzt bei uns im Alltag ganz klar festgestellt, bei uns wird sich aufgrund dieses Zeitdrucks, aufgrund dieses Stress und der Tatsache, dass wir eigentlich unsere Patienten eigentlich recht schnell durchschleusen, dass da oft auf Vorurteile und Stereotypen zurückgegriffen wird und dann wird einfach gesagt: ‚Okay, ja, südländischer Patient, Morbus mediterraneus, machen wir einfach Analgesie und schickt die wieder nach Hause.‘“* | *“Well, I have now clearly noticed in our everyday life that due to this time pressure, due to this stress and the fact that we get our patients through quite quickly, we often resort to prejudices and stereotypes and then one simply says: ‘Okay, yes, Mediterranean patient, just morbus mediterraneus, let's just do analgesia and send them back home.’”* | HCP 25, doctor, inpatient, with migrant background |
| **Challenge related to situational factors** | | | |
| **Challenge ‘Planning and controlling the current workload in outpatient care’** | | | |
| 14 | *Da darf man aber nicht vergessen, Kassenarztpraxis bedeutet ja auch Fünf-Minuten-Medizin im Schnitt. Ich kann mich also jetzt nicht mit einem jungen Mann hinsetzen, der sich mit diesem Problem* [Erektionsprobleme] *vorstellt, und sagen, jetzt nehme ich mir eine halbe Stunde für den Zeit und höre mal genau nach, wo denn nun das Problem ist. Dann würde ja das Wartezimmer überquellen. Das geht nicht“* | *“But you shouldn't forget that a doctor's practice also means an average of five minutes of medicine. So now I can't sit down with a young man who presents this problem* [erectile dysfunction] *and say, now I take half an hour of time and listen exactly where the problem is. Then the waiting room would overflow. That’s not possible“* | HCP 17, doctor, outpatient, without migrant background |
| 15 | *„Also, ja, also ich dachte, es gibt sicherlich einen Unterschied. Also ich habe ja eine reine Terminpraxis, so eine Bestellpraxis als Psychotherapeut und insofern habe ich schon mal nicht das Problem, dass Patienten einfach so daherkommen, ja. Das passiert mir viel weniger“* | *“So, yes, so I thought, there is certainly a difference. I have a purely appointment-based practice, an order-based practice as a psychotherapist, and so I don't have the problem that patients just come along, yes. That happens to me much less.”* | HCP 16, psychologist, with migrant background |
| **Inductive categories ‘Challenges and applied solutions related to the personal factor (shared) migrant background’** | | | |
| **Challenge ‘Ad hoc interpreting outside one’s own treatment situation’** | | | |
| 16 | *“Ich meine, ich komme jetzt auch aus einer Region, da spricht man jetzt kein astreines Türkisch. Das ist schon ziemlicher Slang* (…) *Das wollte ich dann irgendwann nicht mehr gemacht haben und ich fand es eher belastend und das waren häufig Sachen dann so zwischen Tür und Angel irgendwie und dafür wollte ich dann nicht geradestehen.”* | *"I mean, I come from a region where people don't speak pure Turkish. That's quite a slang (…) At some point I didn't want to do that anymore and I found it rather burdening and things often happened between door and hinge, and I didn't want to be held responsible for it.”* | HCP 28, nurse, inpatient, with migrant background |
| **Applied solution ‘Refusal of interpreting for others or providing treatment in native language** | | | |
| 17 | *„Ich habe viele türkische Kollegen, die das ähnlich handhaben und da auch gar nicht dann Türkisch sprechen und sagen, okay, du* [der Patient] *bist halt im deutschen Krankenhaus, dann musst du das halt auch auf Deutsch irgendwie regeln“* | *“I have many Turkish colleagues who handle this similarly and don't speak Turkish at all and say, okay, you* [the patient] *are in a German hospital, then you have to sort it out in German somehow”* | [HCP 30, doctor, inpatient, with migrant background] |
| **Applied solution ‘List of staff who speak foreign languages’** | | | |
| 18 | *„Wir haben zum Beispiel eine Fremdsprachenliste, die in der Klinik bekannt war. Im Grunde genommen war da jede Nation vertreten (…) Ja, das war von der Qualität her immer sehr unterschiedlich, je nachdem wer* [vom Personal] *dann auch tatsächlich dann zum Übersetzen gekommen ist“* | *“*W*e have, for example, a list of foreign languages spoken by the staff that was known in the clinic. Basically, every nation was represented* (…) *Yes, the quality was always very different, depending on who* [of the staff] *actually came to translate.”* | [HCP 27, nurse, inpatient, with migrant background] |
